# Exploring the C-X…π Halogen Bonding Motif: An Infrared and Raman Study of the Complexes of CF_3_X (X = Cl, Br and I) with the Aromatic Model Compounds Benzene and Toluene

**DOI:** 10.3390/molecules18066829

**Published:** 2013-06-10

**Authors:** Nick Nagels, Dieter Hauchecorne, Wouter A. Herrebout

**Affiliations:** Department of Chemistry, University of Antwerp, Groenenborgerlaan 171, B-2020 Antwerp, Belgium

**Keywords:** halogen bonding, C-X···π interactions, infrared spectroscopy, Raman spectroscopy, cryosolutions

## Abstract

The formation of halogen bonded complexes formed between the trifluorohalomethanes CF_3_Cl, CF_3_Br and CF_3_I and the Lewis bases benzene and toluene at temperatures below 150K was investigated using FTIR and Raman spectroscopy. Experiments using liquid krypton as solvent show that for both CF_3_Br and CF_3_I substantial fractions of the monomers can be involved in 1:1 complexes. In addition, weak absorptions illustrating the formation of 2:1 complexes between CF_3_I and benzene are observed. Using spectra recorded at temperatures between 120 and 140 K, observed information on the relative stability was obtained for all complexes by determining the complexation enthalpies in solution. The resulting values for CF_3_Br**·**benzene, CF_3_I**·**benzene and (CF_3_I)_2_**·**benzene are −6.5(3), −7.6(2) and −14.5(9) kJ mol^−1^. The values for CF_3_Br**·**toluene and CF_3_I**·**toluene are −6.2(5) and −7.4(5) kJ mol^−1^. The experimental complexation enthalpies are compared with theoretical data obtained by combining results from MP2/aug-cc-pVDZ(-PP) and MP2/aug-cc-pVTZ(-PP) *ab initio* calculations, from statistical thermodynamical calculations and from Monte Carlo Free Energy Perturbation simulations. The data are also compared with results derived for other C-X···π halogen bonded complexes involving unsaturated Lewis bases such as ethene and ethyne.

## 1. Introduction

Because of their role in biomolecular systems, protein-ligand binding and in supramolecular chemistry in general, the nature of C-X···Y halogen bonded interactions with X = Cl, Br or I has attracted widespread interest of chemists and biochemists [1,2,3]. Apart from interactions where the donor atom is an oxygen, a nitrogen or a sulphur atom, an important role is assigned to the interactions of organic halogen atoms with aromatic amino acid side chains [4,5], the relative importance derived from a recent survey of protein databases being in the order of 53%, 9% and 5% for C-X···O, C-X···N and C-X···S interactions, respectively, and up to 33% for C-X···π interactions [4].

Although the relative importance of various C-X···π interactions is now well recognized, experimental and theoretical data related to the nature of the actual interaction remains rather scarce, the experimental data for aromatic π systems being limited to XRD studies of supramolecular assemblies [6,7,8] and a ^19^F-NMR study of the interactions appearing between toluene and the perfluoroiodopropanes 1-C_3_F_7_I [9].

To deliver systematic data allowing the study of the nature of the weak halogen bonding and their role in various applications, we have recently initiated an infrared and Raman spectroscopic study of halogen bonded complexes formed in cryogenic solutions. The halogen donors used in this study involve, amongst others, the trifluorohalomethanes CF_3_X, with X = Cl, Br and I. The Lewis bases examined so far are dimethyl ether [10], trimethylamine [11] and dimethyl sulfide [12]. In addition, weak C-X···π halogen bonded complexes involving the isolated π system in the alkenes ethene and propene were reported [13]. The cryosolutions used create a weakly interacting environment that, combined with low temperatures, leads to small band widths in the spectra and that facilitates the detection of bands due to complexes that are only slightly shifted from the corresponding monomer modes, as is often the case for weak complexes. Because the cryosolutions are in thermodynamical equilibrium, the solutions also allow the determination of experimental data on the relative stability of the complexes observed.

Triggered by the recent experimental observation of the complexes of the trifluorohalomethanes with ethene and propene, and by the aim at expanding further our knowledge of C-X···Y halogen complexes, we have initiated an infrared and Raman spectroscopic study of the complexes formed between the aromatic model compounds benzene and toluene and the trifluorohalomethanes CF_3_Cl, CF_3_Br and CF_3_I. The experimentally obtained data and the results of the *ab initio* and Monte Carlo calculations, supporting the experimental study, are reported below.

## 2. Results and Discussions

### 2.1.* Ab Initio* Calculations

**Figure 1:**
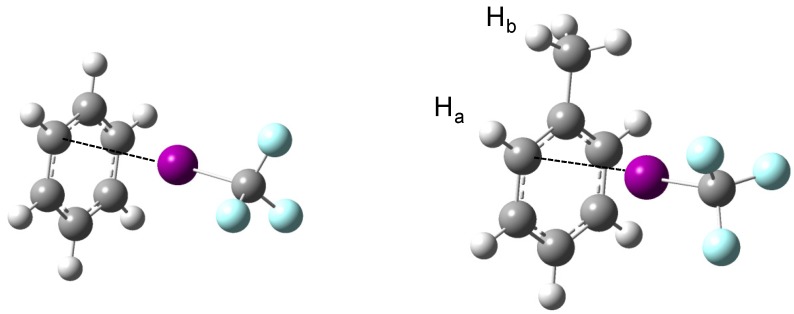
MP2/aug-cc-pVDZ(-PP) equilibrium geometries of the complexes of CF_3_I with benzene and toluene. Small changes observed for the complexes with CF_3_Br and CF_3_Cl are discussed in the text.

The MP2/aug-cc-pVDZ(-PP) optimized geometries, of C_1_ symmetry, of the complexes of CF_3_X (with X = Cl, Br, I) with benzene and toluene are shown schematically in Figure 1. The structural data describing the relative orientation of the monomers, and those related to the most pronounced perturbations are summarized in Table 1 (benzene) and Table 2 (toluene). For both benzene and toluene the equilibrium geometry is characterized by a localized structure [14] in which the halogen atoms are not bonded towards the center of the aromatic ring but are interacting with a single carbon atom. The observation of localized structures is contradictory to the structural data of Lu *et al*. [15] who reported a delocalized complex as the preferred geometry for isolated complexes of benzene with simple halogen containing hydrocarbons such as chlorotrifluoromethane. 

**Table 1:** MP2/aug-cc-pVDZ(-PP) bond lengths, in Å, and bond angles, in degrees, for the complexes of CF_3_Cl, CF_3_Br and CF_3_I with benzene*^a^*.

	CF_3_Cl·benzene		CF_3_Br·benzene		CF_3_I·benzene	
CF_3_X						
*r*CF	1.3450	(0.0024)	1.3464	(0.0024)	1.3497	(0.0028)
*r*CX	1.7510	(−0.0039)	1.9110	(−0.0013)	2.1423	(0.0001)
*θ*XCF	110.72	(0.24)	110.80	(0.26)	111.01	(0.29)
Benzene *^b^*						
*r*CC	1.4072	(0.0006)	1.4077	(0.0011)	1.4084	(0.0018)
*r*CH	1.0933	(0.0001)	1.0934	(0.0002)	1.0935	(0.0003)
*r*Cπ	1.4071	(0.0005)	1.4073	(0.0007)	1.4076	(0.0010)
*θ*CCC	119.99	(−0.01)	119.99	(−0.01)	119.98	(−0.02)
*θ*HCC	120.01	(0.01)	120.01	(0.01)	120.01	(0.01)
Intermolecular *^c^*						
*r*X…Cπ	3.48		3.45		3.50	
*θ*CX…Cπ	173.07		174.39		174.64	

*Notes*: ^a.^The values in brackets are the changes induced by the complexation. The X^…^Cπ interatomic distances are obtained by calculating the distance between the interacting halogen atom and nearest carbon atom of the benzene ring; *^b^*^.^Structural data for the C and H-atoms nearest to the CF_3_X moiety, π refers to the centre of the benzene ring; *^c^*^.^C_π_ refers to the carbon atom of the benzene ring nearest to the CF_3_X moiety.

**Table 2:** MP2/aug-cc-pVDZ(-PP) bond lengths, in Å, and bond angles, in degrees, for the complexes of CF_3_Cl, CF_3_Br and CF_3_I with toluene*^a^*.

	CF_3_Cl·toluene		CF_3_Br· toluene		CF_3_I· toluene	
CF3X						
*r*CF	1.3450	(0.0024)	1.3465	(0.0025)	1.3499	(0.0030)
*r*CX	1.7519	(−0.0030)	1.9121	(−0.0002)	2.1435	(0.0013)
*θ*XCF	110.71	(0.23)	110.78	(0.24)	111.03	(0.31)
Toluene *^b^*						
*r*C=C	1.4068	(−0.0002)	1.4079	(0.0009)	1.4087	(0.0017)
*r*C-C_methyl_	1.5112	(−0.0005)	1.5113	(−0.0004)	1.5112	(−0.0005)
*r*CH_a_	1.0946	(0.0003)	1.0945	(0.0002)	1.0947	(0.0004)
*r*CH_b_	1.1000	(0.0008)	1.0996	(0.0004)	1.0996	(0.0004)
*r*Cπ	1.3993	(0.0006)	1.3994	(0.0007)	1.3996	(0.0009)
*θ*CCC	120.06	(0.01)	120.03	(−0.02)	120.01	(−0.04)
*θ*HCC	119.67	(−0.01)	119.69	(0.01)	119.70	(0.02)
Intermolecular *^c^*						
*r*X…Cπ	3.41		3.38		3.43	
*θ*CX…Cπ	168.80		171.47		173.19	

*Notes*: ^a.^The values in brackets are the changes induced by the complexation. The X^…^Cπ interatomic distances are obtained by calculating the distance between the interacting halogen atom and the nearest carbon atom of the toluene ring; *^b.^*Structural data for the C and H-atoms nearest to the CF_3_X moiety, π refers to the centre of the toluene ring; *^c.^*C_π_ refers to the carbon atom of the toluene ring nearest to the CF_3_X moiety.

The observation of localized equilibrium geometries in the current study is in line with the observation of localized and semi-localized halogen bonded interactions in the crystal structures of bromine and iodine derivatives of trityl alcohols reported by Shishkin [14]. The good agreement, however, might be biased by the fact that the location of the halogen atom as observed in the crystal structures does not immediately reflect the intrinsic properties of the interactions but rather may be dominated by crystal packing effects. Therefore, the theoretical data should ideally be compared with experimental data obtained for isolated complexes as they appear in the gas phase. Because such studies have not yet been reported in literature, this analysis was considered far from straightforward. However, it should be mentioned that Fourier-transform microwave spectroscopic experiments for the benzene·ClF complex lead to a delocalized structure, in which the chlorine atom was pointing towards the centre of the π cloud [16].

In view of the above, it is of interest to note that for the complexes with toluene, all calculations lead to an equilibrium structure in which the X-atom is pointing towards the *ortho* carbon atom C_2_, and that for none of the halogen donors studied, secondary minima on the PES could be located for the *ipso*, *meta* or *para* carbon atoms C_1_, C_3_ or C_4_. For the complexes of toluene with CF_3_Br and CF_3_I, the geometry optimizations also lead to eclipsed orientation of the methyl group in which one hydrogen atom is situated in the plane of the aromatic ring and the other two are situated on both sides of the plane. These results contrast with the data for CF_3_Cl, suggesting that in this complex the methyl group is rotated by 90 degrees. The exact nature of the latter changes is not fully understood, but most probably is related to the nearly free rotation of the methyl group in monomer toluene and by the fact that, although it is significantly weaker, the complex with CF_3_Cl can lead to shorter C-H···F distances and thus can give rise to weak secondary interactions, which compensate the barrier of rotation.

To shed some light on the differences between localized, semi-localized and delocalized geometries for the complexes of benzene, additional geometry optimizations were initiated in which the interacting halogen atom was forced towards the centre of a carbon-carbon bond or towards the centre of the benzene moiety. The resulting energy differences between the localized and delocalized geometries were found to be in the order of 0.1 to 0.7 kJ mol^−1^. The largest differences between the localized and semi-localized geometries were found to be in the order of 0.05 kJ mol^−1^. All these values are smaller than the value of kT, which for a typical experiment in LKr is in the order of 1.0 to 1.4 kJ mol^−1^. The results therefore suggest that in the cryosolutions studied, the halogen donor molecule will undergo large amplitude motions which, in principle, could lead to an average delocalized geometry with C_6_ symmetry [17].

The MP2/aug-cc-pVDZ(-PP) complexation energies for the different complexes studied, the MP2/aug-cc-pVTZ(-PP) values obtained by performing additional single point energy calculations, and the ratio of the interatomic distances to the sum of the Van der Waals radii are summarized in Table 3. It can be seen that expanding the basis set slightly stabilizes most of the complexes, the differences typically being in the order of 2% to 8%. The general trends showing a significant increase from CF_3_Cl towards CF_3_I are in line with the generally accepted ideas suggesting a significant increase in size and depth of the respective σ-holes [18]. The observed increase in stability when passing from benzene to toluene is in line with the electron donating character of the additional methyl group [13].

**Table 3:** Intermolecular distance X^…^Cπ *^a^*, in Å, ratios of the interatomic distances and sum of the respective Van der Waals radii *^b^*, and MP2/aug-cc-pVD(T)Z(-PP) complexation energies ΔE, in kJ mol^−1^, for the complexes of benzene and toluene with CF_3_X (X = Cl, Br, I).

	CF_3_Cl·benzene	CF_3_Br·benzene	CF_3_I·benzene
*r*X…π	3.48	3.45	3.50
sumvdW	3.50	3.65	3.85
ratio	0.99	0.95	0.91
ΔE (DZ)	−11.5	−14.6	−17.9
ΔE (TZ)	−12.4	−15.0	−18.6
	CF3Cl·toluene	CF3Br·toluene	CF3I·toluene
*r*X…π	3.41	3.38	3.43
sumvdW	3.50	3.65	3.85
ratio	0.97	0.93	0.89
ΔE (DZ)	−13.3	−17.1	−21.2
ΔE (TZ)	−13.9	−17.2	−21.7

Notes: *^a.^*The X^…^Cπ interatomic distances are obtained by calculating the distance between the interacting halogen atom and nearest carbon atom of the toluene ring;*^b.^*The values for the van der Waals radii used to calculate sumvdWare 1.70 Å for C, 1.80 Å for Cl, 1.95 Å for Br and 2.15 Å for I.

The MP2/aug-cc-pVDZ(-PP) harmonic vibrational frequencies and infrared intensities of the monomers and complexes are presented in Table S1, Table S2 and Table S3 (benzene) and Table S4, Table S5 and Table S6 (toluene). The data required to rationalize the experimentally observed complexation shifts is presented in Table 4 and Table 5, respectively. To facilitate comparison with calculated and observed frequencies reported in literature data, the normal modes of toluene were split into a group which describes the modes in the phenyl group and a smaller group of 6 describing the methyl vibrations. Following the ascent in symmetry approach, the modes localized in the phenyl group are numbered from 1 to 30, while the methyl vibrations are numbered from 31 to 36 [19]. 

**Table 4:** Selected harmonic MP2/aug-cc-pVDZ(-PP) vibrational frequencies, in cm^−1^, and infrared intensities, in km mol^−1^, for the halogen bonded complexes of benzene and CF_3_Cl, CF_3_Br and CF_3_I, respectively.

Mode	ν_monomer_	IR intensity	ν_complex_	IR intensity	Δν
CF_3_Cl					
*ν_1_*	1097.0	469.7	1098.8	558.7	1.8
*ν_3_*	480.3	0.1	481.5	1.1	1.2
*ν_4_*	1192.2	282.1	1182.6	214.4	−9.6
Benzene					
*ν_2_*	1007.1	0.0	1006.3	1.0	−0.8
*ν_4_*	678.4	115.9	680.6	128.7	2.2
CF_3_Br					
*ν_1_*	1078.6	496.1	1080.0	581.9	1.4
*ν_3_*	361.5	0.0	360.4	1.0	−1.1
*ν_4_*	1178.8	254.7	1169.8	243.6	−9.0
Benzene					
*ν_2_*	1007.1	0.0	1005.9	1.6	−1.2
*ν_4_*	678.4	115.9	681.9	133.4	3.5
CF_3_I					
*ν_1_*	1060.0	546.9	1063.4	625.2	3.5
*ν_3_*	295.1	0.3	293.3	2.6	−1.8
*ν_4_*	1162.0	227.6	1151.8	222.2	−10.2
Benzene					
*ν_2_*	1007.1	0.0	1005.3	3.1	−1.8
*ν_4_*	678.4	115.9	683.5	141.6	5.1

Because of the anticipated large amplitude motions in the complexes the standard harmonic vibrational frequencies deduced for the localized structures might not yield reliable predictions for the complexation shifts. Close inspection of the vibrational modes localized in the interacting monomers and of the complexation shifts derived from them, however, show that only little differences are observed between the vibrational frequencies derived for the different localized and delocalized geometries. As a consequence, is seems appropriate to assume that the vibrational frequencies and complexation shifts derived from the localized geometry can safely be used in all further studies.

**Table 5:** Selected harmonic MP2/aug-cc-pVDZ(-PP) vibrational frequencies, in cm^−1^, and infrared intensities, in km mol^−1^, for the halogen bonded complexes of toluene and CF_3_Cl, CF_3_Br and CF_3_I, respectively.

Mode	ν_monomer_	IR intensity	ν_complex_	IR intensity	Δν
CF3Cl					
*ν_1_*	1097.0	469.7	1097.9	566.5	1.0
*ν_3_*	480.3	0.1	481.2	1.6	0.9
*ν_4_*	1192.2	282.1	1183.0	260.4	−9.2
Toluene					
*ν_4_*	1641.4	5.9	1639.0	5.3	−2.4
CF3Br					
*ν_1_*	1078.6	496.1	1079.6	596.5	1.0
*ν_3_*	361.5	0.0	359.5	1.3	−2.0
*ν_4_*	1178.8	254.7	1169.7	240.9	−9.1
Toluene					
*ν_4_*	1641.4	5.9	1638.3	4.9	−3.1
CF3I					
*ν_1_*	1060.0	546.9	1063.4	640.9	3.5
*ν_3_*	295.1	0.3	292.3	3.2	−2.8
*ν_4_*	1162.0	227.6	1151.1	220.2	−10.9
Toluene					
*ν_4_*	1641.4	5.9	1637.1	4.6	−4.3

**Figure 2:**
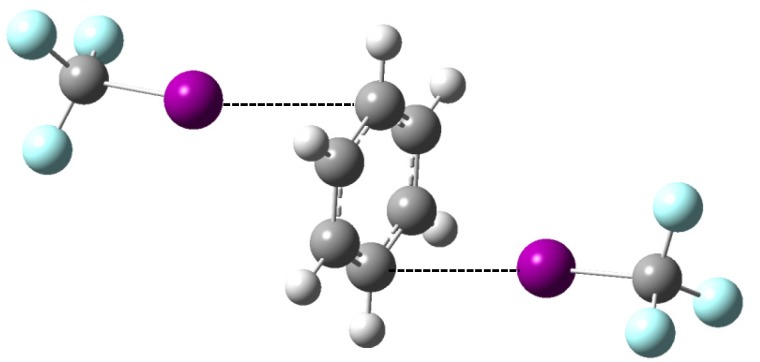
MP2/aug-cc-pVDZ(-PP) equilibrium geometry of the 2:1 complex involving two CF_3_I molecules and a single benzene molecule.

It will be shown below that evidence for the formation of a 2:1 complex, containing two molecules CF_3_I and a single benzene moiety, is found in the experimental studies. Theoretical information on this 2:1 complex was, therefore, also derived. The resulting equilibrium geometry is shown in Figure 2. Comparison of the MP2/aug-cc-pVTZ-PP complexation energies for the 1:1 and 2:1 complexes, given in Table 6, reveals that the value of the 2:1 complex is a mere 2% smaller than twice the value of the 1:1 complex. This suggests that, analogous to the 2:1 halogen bonded complexes observed in previous studies [10,12,13], the binding of the second CF_3_I unit weakens the first halogen bond and vice versa. The occurrence of an anti-cooperative effect is also illustrated by the characteristic frequencies for the 1:1 and 2:1 complexes and those of the monomers, summarized in Table 7, showing that the shift for modes in the benzene moiety for the 2:1 complex is larger than that for the 1:1 complex, while for the modes in the competing CF_3_I moieties the opposite trend is observed. 

**Table 6:** Transformation of the *ab initio* MP2/aug-cc-pVTZ(-PP) complexation energies, ∆*E*(calc), to the standard complexation enthalpies in LKr by applying thermal and solvent corrections. For completeness, the experimental standard complexation enthalpies are also included. All values are given in kJ mol^−1^.

	Benzene				Toluene		
	CF_3_Cl	CF_3_Br	CF_3_I	2CF_3_I	CF_3_Cl	CF_3_Br	CF_3_I
∆*E*(calc)	−12.4	−15.0	−18.6	−35.0	−13.9	−17.2	−21.7
∆*H* (vap,calc)	−10.3	−12.9	−16.5	−30.8	−11.8	−15.1	−19.6
∆*H* (LKr,calc)	−7.5	−9.4	−12.4	−23.6	−8.5	−10.9	−15.1
Experimental							
∆*H* (LKr)		−6.5	−7.6	−14.5		−6.2	−7.4

**Table 7:** Characteristic vibrational frequencies and complexation shifts (values in brackets), in cm^−1^, for the 1:1 and 2:1 complexes of CF_3_I with benzene. The experimental data refer to a solution in liquid krypton, at 120 K. The calculated values were derived from the MP2/aug-cc-pVDZ-PP frequencies.

		Calculated	Experiment
*ν*_4_	benzene	678.4	674.8
	CF3I·benzene	683.5 (5.1)	679.4 (4.6)
	2CF3I·benzene	688.4 (10.0)	683.2 (8.4)
*ν*_4_	CF_3_I	1162.0	1175.6
	CF3I·benzene	1151.8 (−10.2)	1165.3 (−10.3)
	2CF3I·benzene	1154.4 (−7.6)	

It should be mentioned here that so far no experimental evidence was found for the formation of a 2:1 complex between CF_3_I and toluene. As a consequence, no calculations for this type of complex were pursued.

### 2.2. Statistical Thermodynamics and Monte Carlo-Free Energy Perturbation Simulations

In a first step towards the comparison of theory and experiment, vapor phase complexation enthalpies ∆*H*(vap,calc) were predicted by correcting the MP2/aug-cc-pVTZ(-PP) complexation energies ∆*E*(calc) for thermal and zero-point vibrational contributions. To this end, statistical thermodynamical calculations were performed using a rigid rotor/harmonic oscillator model. For all calculation the temperature was set at 130 K, *i.e.*, at the midpoint of the temperature interval used during the experimental study. Subsequently, the value for the complexation enthalpy in LKr, ∆*H*(LKr,calc), was estimated using Monte Carlo Free Energy Perturbation simulations. To this end, for each species, the solvation Gibbs energies in LKr were estimated at 6 different temperatures, varying from 94 to 154 K, at a pressure of 28 bar, and the enthalpy of solvation Δ_sol_*H* was extracted using the expressions Δ_sol_*H* = Δ_sol_*G* + *T*Δ_sol_*S* and Δ_sol_*S* = −(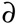
Δsol*G*/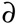
*T*)p. The resulting values for the vapor phase complexation enthalpies and the corresponding values for the solution in LKr are given in Table 6.

### 2.3. Vibrational Spectra

The vibrational spectra of the trifluorohalomethanes CF_3_Cl, CF_3_Br and CF_3_I and of benzene dissolved in liquid rare gases have been studied in detail before [10,13,20,21] and are not commented here. The observed frequencies for toluene dissolved in LKr and their assignments, based on literature data for the vapor and liquid phases [19] and on the harmonic vibrational frequencies derived at the MP2/aug-cc-pVDZ and MP2/aug-cc-pVTZ levels, are summarized in Table S7. It can be seen that for almost all vibrations an excellent agreement is found between the experimental and calculated frequencies. A typical exception is found for ν_18_, which in the infrared spectrum gives rise to an intense feature near 695 cm^−1^ and which at the MP2/aug-cc-pVDZ level is calculated to appear as a weak feature, with an infrared intensity of a mere 1.7 km mol^−1^, at 609.8 cm^−1^. A much more reliable prediction leading to a harmonic vibrational frequency of 713.4 cm^−1^ and a calculated infrared intensity of 11.3 km mol^−1^ is obtained at the MP2/aug-cc-pVTZ level. The large deviation for the MP2/aug-cc-pVDZ level and the more reliable value for the MP2/aug-cc-pVTZ level is explained as the result of severe Basis Set Incompleteness Errors [22,23] appearing at the lower level.

To fully characterize the halogen bonded complexes of CF_3_X (with X = Cl, Br and I) with benzene and toluene, in this study infrared and Raman spectra of solutions in LKr containing mixtures of the different halogen donors and acceptors were recorded at temperatures between 120 and 140 K. For the infrared measurements, the mole fractions for the trifluorohalomethanes and the Lewis bases were typically varied between 7.5 × 10^−5^ and 3.4 × 10^−2^ and between 3.8 × 10^−5^ and 1.9 × 10^−3^, respectively. The Raman spectra were recorded using mole fractions between 1.7 × 10^−3^ and 1.3 × 10^−2^ for the mixtures with benzene and between 4.7 × 10^−3^ and 7.1 × 10^−3^ for the mixtures with toluene. Even at relatively high concentrations of the molecules involved, for none of the mixtures with CF_3_Cl, indications for the formation of a C-Cl^…^π halogen bonded complex were observed. The obvious conclusion is that these complexes are too weak to be observed in LKr. The observed frequencies, the assignment and the complexation shifts obtained for the complexes with benzene and with toluene are collected in Table 8 and Table 9, and in Table 10 and Table 11, respectively.

Previous experiments dealing with the complexes of the trifluorohalomethanes with, amongst other, dimethyl ether, dimethyl sulfide and trimethyl amine have shown [10,11,12] that upon complexation with a Lewis base significant shifts can be observed for the CF_3_ symmetric and antisymmetric stretches ν_1_ and ν_4_. In agreement with these results, also in this study significant red shifts, varying between −9.0 and −10.2 cm^−1^ for the complexes with benzene and between −9.1 and −10.9 cm^−1^ for the complexes with toluene, are predicted for the antisymmetric stretches ν_4_. In addition, for the symmetric stretching modes ν_1_ small blue shifts, varying between +1.4 and +3.5 cm^−1^ and between +1.0 and +3.5 cm^−1^, are predicted, respectively. 

**Table 8:** Experimental vibrational frequencies and complexation shifts, in cm^−1^, for the complex of CF_3_Br with benzene dissolved in LKr at 120 K. The *ab initio* complexation shifts given are derived from the MP2/aug-cc-pVDZ-PP harmonic vibrational frequencies.

	Assignment	ν_monomer_	ν_complex_	Δν_exp_	Δν_calc_
CF_3_Br	ν_1_ + ν_2_	1831.2	1831.9	0.7	−1.3
	ν_4_	1198.1	1190.4	−7.7	−9.0
	ν_1_	1075.2			1.4
	ν_2_	759.4	758.5	−0.8	−2.7
	ν_5_	546.3			−0.8
	ν_3_ (^79^Br)	352.1	352.0	−0.1	−1.1
	ν_3_ (^81^Br)	350.3	350.2	−0.1	−1.1
	ν_6_	303.4			0.5
Benzene	ν_1_	3067.8			0.6
	ν_1__5_	3052.2			1.2
	ν_1__2_	3041.6	3042.2	0.6	0.8
	ν_7_ + ν_19_	1955.0	1958.1	3.1	1.2
	ν_11_ + ν_19_	1810.1	1814.1	4.0	3.6
	ν_4_+ ν_11_	1520.2	1525.5	5.3	6.1
	ν_13_	1481.3	1481.2	−0.1	−2.0
	ν_17_	1175.1			−0.1
	ν_14_	1037.6			−0.8
	ν_2_	992.7	992.0	−0.7	−1.2
	ν_4_	674.8	677.4	2.6	3.5

**Table 9:** Experimental vibrational frequencies and complexation shifts, in cm^−1^, for the complex of CF_3_I with benzene dissolved in LKr at 120 K. The *ab initio* complexation shifts given are derived from the MP2/aug-cc-pVDZ-PP harmonic vibrational frequencies.

	Assignment	ν_monomer_	ν_complex_	Δν_exp_	Δν_calc_
CF_3_I	2ν_4_ (*l*_4_=0)	2329.9	2309.9	−20.0	−20.4
	ν_2_ + ν_4_	1909.9	1898.1	−11.8	−13.0
	ν_4_	1175.6	1165.3	−10.3	−10.2
	ν_4_ (^13^C)	1142.7	1132.5	−10.2	−10.2
	ν_1_	1067.4	1070.1	2.7	3.5
	ν_1_ (^13^C)	1040.0	1042.7	2.7	3.5
	ν_2_	740.7			−2.8
	ν_5_	539.9			−1.0
	ν_3_	286.4	285.6	−0.8	−1.8
	ν_6_	266.0			0.3
Benzene	ν_1_	3067.8			0.9
	ν_1__5_	3052.2			1.7
	ν_1__2_	3041.6	3041.1	−0.5	1.1
	ν_7_ + ν_19_	1955.0	1959.6	4.6	1.1
	ν_11_ + ν_19_	1810.1	1816.3	6.2	4.7
	ν_4_+ ν_11_	1520.2	1528.5	8.3	8.6
	ν_13_	1481.3	1480.8	−0.5	−2.9
	ν_17_	1175.1			−0.2
	ν_14_	1037.6	1037.2	−0.4	−1.2
	ν_2_	992.7	991.4	−1.3	−1.8
	ν_4_	674.8	679.4	4.6	5.1

**Table 10:** Experimental vibrational frequencies and complexation shifts, in cm^−1^, for the complex of CF_3_Br with toluene dissolved in LKr at 120 K. The ab initio complexation shifts given are derived from the MP2/aug-cc-pVDZ-PP harmonic vibrational frequencies.

	Assignment	ν_monomer_	ν_complex_	Δν_exp_	Δν_calc_
CF_3_Br	ν_1_ + ν_2_	1831.2			−2.1
	ν_4_	1198.1	1189.8	−8.3	−9.1
	ν_1_	1075.2			1.0
	ν_2_	759.4	758.0	−1.4	−3.1
	ν_5_	546.3			−0.9
	ν_3_ (^79^Br)	352.1	351.5	−0.6	−2.0
	ν_3_ (^81^Br)	350.3	349.7	−0.6	−2.0
	ν_6_	303.4			0.5
Toluene	ν_21_	3092.9	3091.6	−1.3	1.4
	ν_1_	3072.2	3071.8	−0.4	1.3
	ν_2_	3061.1			1.5
	ν_34_	2954.7	2955.0	0.3	−0.5
	ν_31_	2923.1	2922.8	−0.3	−1.9
	ν_15_ + ν_16_	1870.6			4.2
	ν_4_	1607.8	1607.2	−0.6	−3.1
	ν_5_	1497.4	1497.3	−0.1	−1.8
	ν_8_	1031.6	1031.4	−0.2	−0.6
	ν_17_	728.6	731.2	2.6	2.2
	ν_18_	694.5			−4.1
	ν_19_	463.8			−0.3

**Table 11:** Experimental vibrational frequencies and complexation shifts, in cm^−1^, for the complex of CF_3_I with toluene dissolved in LKr at 120 K. The ab initio complexation shifts given are derived from the MP2/aug-cc-pVDZ-PP harmonic vibrational frequencies.

	Assignment	ν_monomer_	ν_complex_	Δν_exp_	Δν_calc_
CF_3_I	2ν_4_ (*l*_4_ = 0)	2329.9	2308.9	−21	−21.8
	ν_2_ + ν_4_	1909.9	1897.1	−12.8	−14.3
	ν_4_	1175.6	1164.8	−10.8	−10.9
	ν_4_ (^13^C)	1142.7	1132.3	−10.4	−10.9
	ν_1_	1067.4	1070.5	3.1	3.5
	ν_1_ (^13^C)	1040.0	1042.5	2.5	3.5
	ν_2_	740.8	738.7	−2.1	−3.4
	ν_5_	539.9			−1.2
	ν_3_	286.4	285.1	−1.3	−2.8
	ν_6_	266.0			0.2
Toluene	ν_21_	3092.9	3091.1	−1.8	1.8
	ν_1_	3072.2	3071.6	−0.6	1.9
	ν_2_	3061.1	3061.3	0.2	2.1
	ν_34_	2954.7	2956.0	1.3	0.1
	ν_31_	2923.1	2922.7	−0.4	−1.1
	ν_15_ + ν_16_	1870.6	1875.9	5.3	5.8
	ν_4_	1607.8	1606.3	−1.5	−4.3
	ν_5_	1497.4	1496.9	−0.5	−2.3
	ν_8_	1031.6	1031.2	−0.4	−0.8
	ν_17_	728.6	731.5	2.9	3.5
	ν_18_	694.5	694.9	0.4	−7.5
	ν_19_	463.8	464.2	0.4	-1.0

**Figure 3:**
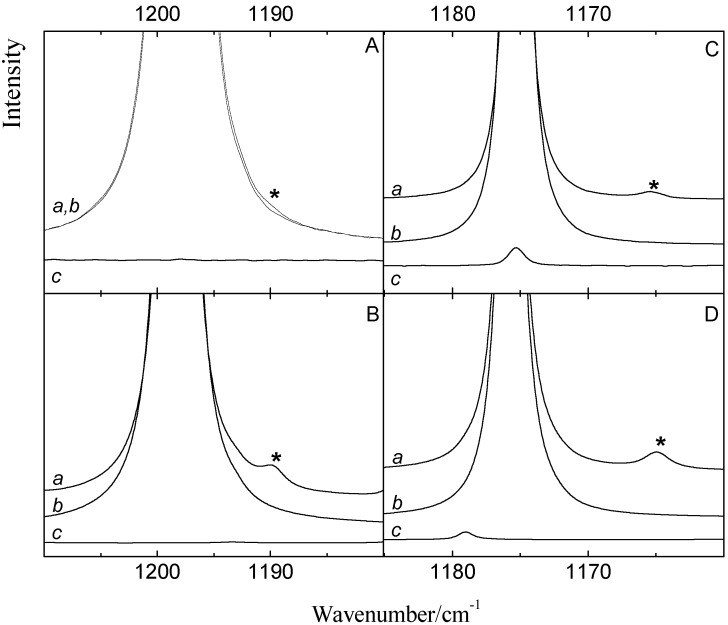
Infrared spectra of the antisymmetric ν_4_ stretching region of CF_3_X (with X = Br, I) for LKr solutions of mixtures of CF_3_Br (panels A and B) or CF_3_I (panels C and D) with benzene (panels A and C) or toluene (panel B and D) at 120 K. In each panel trace *a* represents the mixed solution, while traces *b* and *c* show the solution containing only CF_3_X (with X = Br, I) or the Lewis base, respectively. New bands appearing in the spectrum of the mixtures are marked with an asterisk (*) and are assigned to the 1:1 complex.

Figure 3 shows the ν_4_ spectral regions of solutions in LKr, at 120 K, containing mixtures of benzene with CF_3_Br (panel A) and CF_3_I (panel C) and of toluene with CF_3_Br (panel B) and CF_3_I panel D). To favor complexation with the less soluble Lewis bases, the spectra were recorded for solutions containing a large excess of the trifluorohalomethanes. These conditions, obviously, lead to full absorption in the region of the monomer transitions. 

Comparison of the spectra of the mixtures, shown in trace *a*, with the spectra of the monomers, given in traces *b* and *c*, reveals the presence of weak bands that are assigned to the halogen bonded complexes. Additional bands, only present in the spectra of the mixture, are observed at 1165.3, 1164.8 and 1189.8 cm^−1^ for the complex of CF_3_I with benzene and for the complexes of CF_3_I and CF_3_Br with toluene, respectively. For the mixture of CF_3_Br and benzene in panel A, an extremely weak feature can be envisaged near 1190.4 cm^−1^. The small red shift of −7.7 cm^−1^ deduced by assuming that this feature is related to the halogen bonded complex between benzene and CF_3_Br, and the red shifts of −10.3, −8.3 and −10.8 cm^−1^ observed for the complexes of CF_3_I with benzene and of CF_3_Br and CF_3_I with toluene are in excellent agreement with the calculated values of −9.0, −10.2, −9.1 and −10.9 cm^−1^, given in Table 8, Table 9, Table 10 and Table 11.

Since different concentrations have been used for the benzene and toluene mixtures, the intensities of the complex bands in Figure 3 cannot be used to deduce direct information on the relative stabilities of the different complexes. However, the fact that similar complexation shifts have been observed for the corresponding benzene and toluene complexes, suggests, albeit indirectly, comparable stabilities for the CF_3_Br·benzene and CF_3_Br·toluene halogen bonds and for the CF_3_I·benzene and CF_3_I·toluene halogen bonds.

Apart from the shifts observed for the CF_3_ stretching modes, complexation shifts have also been predicted for the C-X stretching modes. These modes are characterized by a very low infrared intensity in the monomers and, consequently, are preferentially studied using Raman spectroscopy. Careful analysis of the spectra of the mixed solutions and of the spectra of the monomers shows that also for the mixtures studied here, indications for complex bands due to halogen bonded complexes can be observed at a frequency somewhat lower than that of the monomer. The experimental complexation shifts for the C-^79^Br, the C-^81^Br and the C-I stretching modes are in the order of −0.1 to −0.8 cm^−1^ for the complexes with benzene and in the order of −0.6 to −1.3 cm^−1^ for the complexes with toluene. These values are in line with, but somewhat smaller than, the calculated values reported in Table 4 and Table 5. 

**Figure 4:**
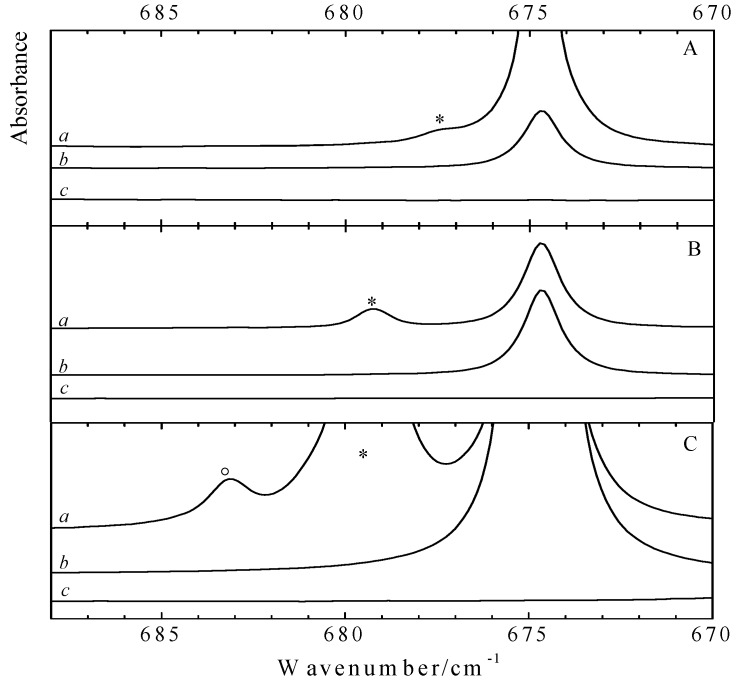
Infrared spectra of the C-H out of plane bending mode ν_4_ (A_2u_) of benzene for LKr solutions of mixtures of CF_3_Br (panel A) or CF_3_I (panels B and C) with benzene at 120K. In each panel trace *a* represents the mixed solution, while traces *b* and *c* show the solution containing only benzene and CF_3_X (with X = Br, I), respectively. New bands appearing in the spectrum of the mixtures are marked with an asterisk (*) and are assigned to the 1:1 complex. Panel C is obtained by using higher concentrations of both CF_3_I and benzene. In this panel, the band marked with a circle (°) is assigned to the 2:1 complex with two molecules CF_3_I and a single molecule benzene.

**Figure 5:**
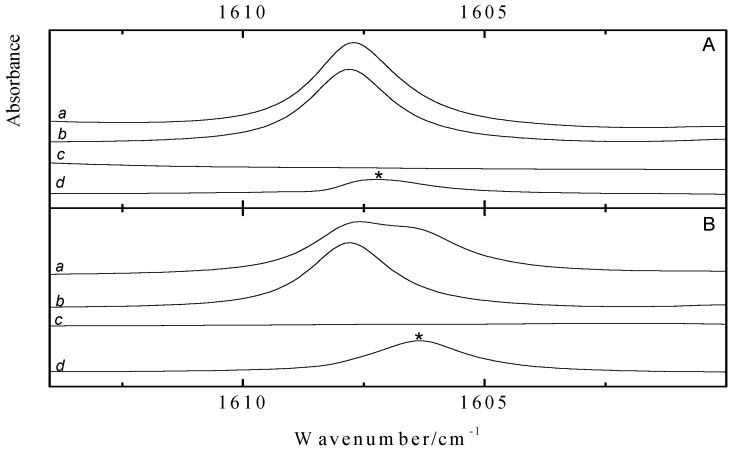
Infrared spectra of the in plane deformation ν_4_ (A_1_) of toluenefor LKr solutions of mixtures of CF_3_Br (panel A) or CF_3_I (panel B) with toluene at 120K. Additional bands are visible in the spectrum of the complex (trace *d*), which is obtained after subtracting the rescaled monomer traces *b* and *c* from the mixture trace *a*. Traces *b* and *c* show the solution containing only toluene and CF_3_X (with X = Br, I), respectively. New bands appearing in thespectrum of the complex are marked with an asterisk (*) and are assigned to the 1:1 complex.

Analysis of the data in Table S1, Table S2, Table S3, Table S4, Table S5 and Table S6 reveals that also for the Lewis bases significant complexation shifts and intensity changes can be expected upon complexation. Typical examples of complex bands observed while analyzing the spectral regions related to the C-H out of plane bending mode ν_4_ (A_2u_) in benzene and to the in plane deformation ν_4_ (A_1_) in toluene are depicted in Figure 4 and Figure 5. The infrared spectra obtained for solutions in LKr, at 120 K, containing mixtures of the Lewis base with CF_3_Br and CF_3_I are shown in panels A and B, respectively. Since the vibrational mode ν_4_ of the monomer benzene has high infrared intensity, with a predicted value of 115.9 km mol^−1^, only low concentrations of benzene could be used to study this region. As a consequence, complexation was forced by using a large excess of the halogen donor. The typical mole fractions used for CF_3_I, CF_3_Br and benzene are 9.1 × 10^−3^, 1.3 × 10^−2^ and 4.9 × 10^−4^. The spectra for the solutions containing benzene and an even larger excess of CF_3_I, with mole fractions close to 9.4 × 10^−5^ and 2.0 × 10^−2^, are given in panel C of Figure 4. The significantly lower infrared intensity of the monomer vibration ν_4_ of toluene, with a calculated value of 5.9 km mol^−1^, allows the usage of higher mole fractions in the order of 1.9 × 10^−3^ which, combined with mole fractions of 9.4 × 10^−5^ and 2.3 × 10^−4^ for CF_3_I and CF_3_Br, result in the spectra shown in Figure 5. As before, the spectra of the mixed solutions are given in trace *a* while the spectra of the corresponding monomers are given in traces *b* and *c*. The bands related to halogen bonded complexes are marked with an asterisk. 

Analysis of the data in the different panels of Figure 4 reveals that for both CF_3_Br and CF_3_I a new band appears on the high frequency side of the 674.8 cm^−1^ monomer vibration. The blue shifts of + 2.6 cm^−1^ for the CF_3_Br complex and + 4.6 cm^−1^ for CF_3_I are in line with the *ab initio* complexation shifts, presented in Table 4, and agree favorably with data reported in earlier studies showing that the C-H out of plane bending mode ν_4_ (A_2u_) in benzene is largely perturbed by C-H proton donors such as halothane, CF_3_CBrClH [20], and sevoflurane, (CF_3_)_2_CHOCH_2_F [21], and other Lewis acids such as hexafluorobenzene, C_6_F_6_ [24].

Apart from the bands assigned to the monomers and to the 1:1 complexes, in the infrared spectra of the mixed solutions containing higher concentrations of benzene and CF_3_I an additional spectral feature at 683.2 cm^−1^ is observed. This band, which in Figure 4C is marked with a circle (°), is observed at lower temperatures and at highly concentrated solutions only. Supported by the results of the *ab initio* calculations and a concentration study, to be discussed below, and by the appearance of similar features for solutions containing benzene and halothane, this band is assigned to the ν_4_ mode of benzene in a 2:1 complex consisting of two CF_3_I molecules and one benzene moiety. 

Analysis of the ν_4_ spectral data of toluene, presented in Figure 5, shows that apart from the monomer transition at 1607.8 cm^−1^, new features due to the same mode in the complex with CF_3_Br and CF_3_I become apparent near 1607.2 and 1606.3 cm^−1^. The presence of these features is confirmed using subtraction procedures in which spectra of the monomers are recorded at exactly the same temperature as that used to study the mixed solution and are rescaled afterwards to correctly reproduce the monomer contributions in spectral regions where monomers and complexes are easily distinguished. The spectra of the mixed solutions, the rescaled spectra of the monomers, and the difference spectrum obtained by subtracting the various spectra are shown in traces *a*, *b* and *c*, and *d*, respectively. The red shifts derived from the data are −0.6 cm^−1^ for the complex with CF_3_Br and −1.5 cm^−1^ for the complex with CF_3_I. These values, again, are in line with the theoretical shifts of −3.1 and −4.3 cm^−1^ and confirm the above observations suggesting that the predicted complexation shifts in almost all cases are strongly overestimated. 

The vibrational frequencies and infrared intensities in Table 4 show that upon complexation with CF_3_I and CF_3_Br, a small red shift is observed for the ring breathing mode of benzene. In addition, a significant increase in infrared intensity, from 0.0 km mol^−1^ up to 1.6 km mol^−1^ for CF_3_Br·benzene and 3.1 km mol^−1^ for CF_3_I·benzene, is encountered. The value of 0.0 km mol^−1^ obtained for monomer benzene is in line with the general selection rules showing that the ring breathing mode located near 993 cm^−1^ is allowed in Raman but forbidden in infrared. The values of 1.6 and 3.1 km mol^−1^ obtained for the complexes with CF_3_Br and CF_3_I illustrate that during complexation, the symmetry restrictions are lifted, and that due to the electric field generated by the halogen donor and by the relatively large polarizability derivatives for the mode under studied, the mode gains some induced infrared intensity. 

**Figure 6:**
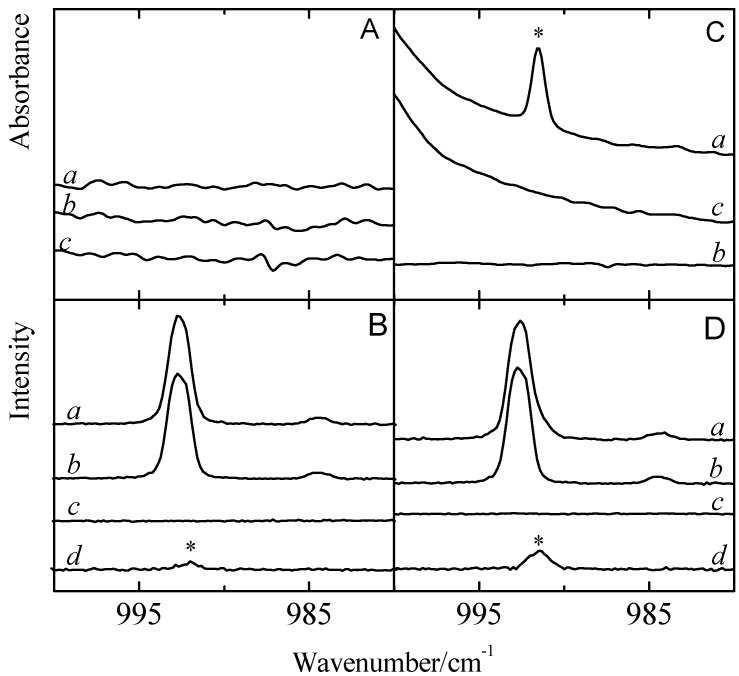
Panels A and C present the infrared spectra and panels B and D the Raman spectra of the so-called ring breathing mode ν_2_ of benzene for solutions with CF_3_Br (panels A and B) and CF_3_I (panels C and D). Trace *a* gives the spectrum of the mixed solution, while traces *b* and *c* are the spectra of the monomer benzene and CF_3_X (with X = Br, I), respectively. Trace *d* is the spectrum of the 1:1 complex, obtained by subtracting the rescaled monomer traces *b* and *c* from the mixture trace *a*. New bands appearing in the spectrum of the complex are marked with an asterisk (*) and are assigned to the 1:1 complex.

To shed light on the appearance of induced spectral features and to further rationalize the behavior of the ring breathing mode in the complexes formed with benzene, special attention was paid to infrared and Raman spectra in the ν_2_ spectral region. Typical spectra obtained for mixed solutions of benzene with CF_3_Br and CF_3_I, and of the monomers involved are summarized in Figure 6. The infrared and Raman data are given in panels A and C, and in panels B and D, respectively. The data for CF_3_Br is shown in panels A and B while the data obtained for CF_3_I is given in panels C and D. In each panel, trace *a* refers to the spectrum of the mixed solution, while traces *b* and *c* are the spectra of monomer benzene and CF_3_X, respectively. Trace *d* is the spectrum of the 1:1 complex, obtained by subtracting the rescaled monomer traces *b* and *c* from the mixture trace *a*.

Inspection of the difference spectra shown in traces *d* of panels B and D shows that for CF_3_Br and CF_3_I, a new band can be observed to appear red-shifted from the 992.7 cm^−1^ monomer transition, by −0.7 and −1.3 cm^−1^, respectively. The frequency of the complex band observed for CF_3_I·benzene is in excellent agreement with that of the induced spectral feature observed in the infrared spectra in panel C, and confirms its assignment. Even for a large excess of CF_3_Br, no counterpart of the 992.0 cm^−1^ complex band observed in the Raman spectra, could be detected in the infrared spectra in panel A. The occurrence of an induced ring breathing mode in the complex with CF_3_I and the lack of such a feature for the complex with CF_3_Br is not fully understood. The observations, however, are in line with the differences in infrared intensity, suggesting that the induced ring breathing mode in the complex with CF_3_I is almost twice as large as that in the complex with CF_3_Br, and with the above observations showing that even when a large excess of CF_3_Br is used, the equilibrium concentrations of the complexes formed remain rather small. The changes in induced infrared intensity, in a first approximation, can be correlated with the large differences in dipole moment between CF_3_I and CF_3_Br and with the level of charge transfer present, giving rise to an approximate penetration depth [25] and an X^…^π intermolecular distance of −0.20 and −0.35 Å, and of 3.45 and 3.50 Å, respectively. 

### 2.4. Stoichiometry

The stoichiometry of the observed complexes was confirmed by isothermal infrared studies in which the spectra of mixed solutions, containing different concentrations of the monomers involved, are recorded at a specific temperature, e.g., 120 K. The data analysis is based upon the fact that under chemical equilibrium conditions the integrated intensity of a complex band under study 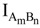 is linearly related to the product of the *m*^th^ power of the monomer band area *I_A_* and the *n*^th^ power of the monomer band area *I_B_*. By plotting the complex band area *I_complex_* versus the products of the monomer band areas (*I_A_*)^x^(*I_B_*)^y^ for various integer values of *x* and *y*, the stoichiometry of the complex can be deduced.

Typical values for the goodness of fit parameter χ^2^ for the mixed solutions containing CF_3_I and benzene, obtained by using the integrated band areas for the 679.4 cm^−1^ complex band and the 1909.9 and 3067.8 cm^−1^ monomer bands, are, with the stoichiometry given in between brackets, 0.0003 (1:1), 0.0032 (2:1) and 0.0266 (1:2). The small value obtained for the proposed 1:1 stoichiometry, and the significantly larger values for the other proposed stoichiometries, confirm the above assignments. 

**Figure 7:**
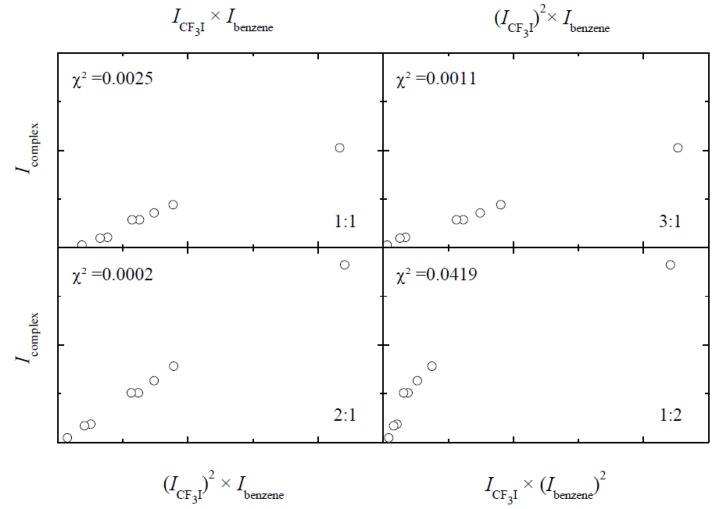
Typical results obtained for the concentrations studies supporting the assignment of the 683.2 cm^−1^ band observed in spectra of mixed solutions in LKr containing CF_3_I and benzene to a complex with 2:1 stoichiometry.

The results of a similar analysis for the band at 683.2 cm^−1^ observed in the mixtures of CF_3_I and benzene are presented in Figure 7. The χ^2^ values obtained for the different stoichiometries are 0.0025 (1:1), 0.0002 (2:1) and 0.0011 (1:2), and support the assignment of the 683.2 cm^−1^ band to a 2:1 complex, in which two molecules of CF_3_I interact with a single benzene molecule. 

Similar values of χ^2^ confirming the stoichiometry of the different complexes observed in the spectra of mixed solutions containing both CF_3_X (with X = Br, I) and benzene or toluene were also obtained for other bands reported in Table 8, Table 9, Table 10 and Table 11. The results of these analyses are considered straightforward and are not reported in detail here.

### 2.5. Relative Stability

Standard complexation enthalpies for the various 1:1 and 1:2 complexes were obtained from temperature studies in which the infrared spectra of mixed solutions were studied as a function of temperature. The mole fractions used typically varied between 7.5 × 10^−5^ and 3.4 × 10^−2^ for the halogen donor and between 3.8 × 10^−5^ and 1.9 × 10^−3^ for the halogen acceptor. The temperature ranges used were determined by the solubility of benzene and toluene in LKr, and typically spanned the range from 120 to 140 K. The complexation enthalpies were derived by using the Van’t Hoff relation which establishes a linear relation between the inverse temperature and the logarithm of the appropriate intensity product 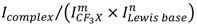. The slopes of these relations equal 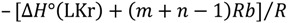 where *b* is a correction factor to account for the changes in solvent density upon temperature variation [26].

**Figure 8:**
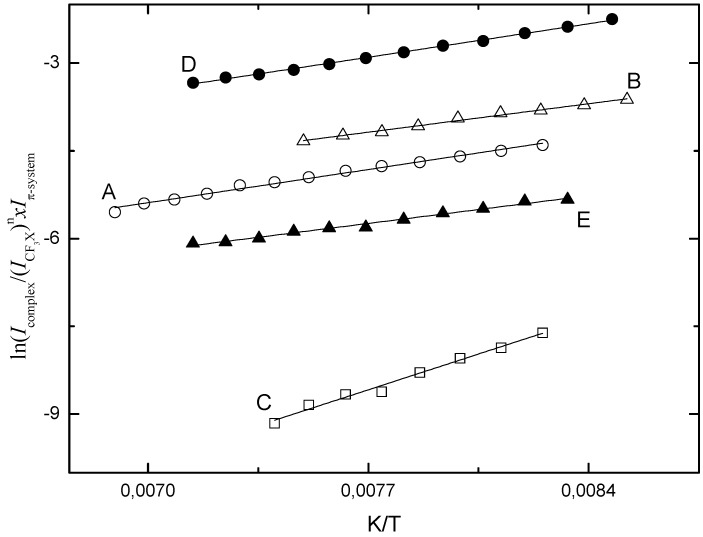
Typical Van’t Hoff plots obtained for the halogen bonded complexes observed in liquid krypton: (**A**) CF_3_I·benzene, (**B**) CF_3_Br·benzene, (**C**) (CF_3_I)_2_.benzene, (**D**) CF_3_I·toluene and (**E**) CF_3_Br·toluene.

Typical Van’t Hoff plots obtained for the different complexes studied are shown in Figure 8. The average complexation enthalpies for the 1:1 complexes, obtained by analyzing and averaging out the data for a series of solutions and by correcting the slopes of the different regression lines for density variations in the temperature intervals used, are −6.5(3) kJ mol^−1^ for CF_3_Br·benzene, −7.6(2) kJ mol^−1^ for CF_3_I·benzene, −6.2(5) kJ mol^−1^ for CF_3_Br·toluene and −7.4(5) kJ mol^−1^ for CF_3_I·toluene. For completeness, Figure 8 also contains a typical Van’t Hoff plot obtained for the 2:1 complex of CF_3_I and benzene. The cumulative complexation enthalpy for this complex, obtained by assuming the complexation equilibrium, 2 CF_3_I + benzene 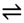 (CF_3_I.)_2_benzene, is −14.5(9) kJ mol^−1^.

Comparison of the experimental complexation enthalpies and the theoretical values summarized in Table 6 shows that the different complexes with benzene and toluene are characterized by very similar complexation enthalpies and that the increase of the predicted values of the relative stability, due to the additional methyl group, are far from reproduced. The experimental observation that the extra methyl group has little or no effect on the relative stability might be somewhat surprising, but in fact is in line with the above observations showing that the complexes with benzene and toluene lead to very similar complexation shifts in the experimental spectra. 

The experimental and predicted values in Table 6 further illustrates that for both aromatic model compounds, the predicted complexation enthalpies are largely overestimated, the differences between experiment and theory being 45% to 63% for the complexes with benzene, and between 76% and 104% for the complexes with toluene. These trends are in line with earlier observations showing that also for other complexes involving the aromatic model compounds including, amongst others, the complexes of benzene with the anesthetics halothane [20] and sevoflurane [21], MP2/aug-cc-pVTZ (-PP) *ab initio* calculations tend to seriously overestimate the complexation energies and the corresponding complexation enthalpies. The overestimation, most likely, is due to imperfections related to the MP2 second order perturbation approach used. To fully rationalize the nature of these effects, it is considered of great interest to compare the experimental values for the different complexes reported with high level *ab initio* calculations in which the complexation energy of the complexes is evaluated at the CCSD(T)/CBS level, or using the recently developed post-MP2 methods or MP2 variants such as scaled MP3 or scaled spin-component MP2 methods [27,28,29,30]. A full analysis of the results obtained using these methods for all hydrocarbons studied so far was deemed beyond the scope of the current study, and will be reported separately in a later phase.

It is of interest to note here that, although the calculations strongly overestimate the stability of the individual complexes, the experimentally observed anti-cooperative effect yielding an experimental complexation enthalpy for the individual C-X^...^π halogen bonds in the 2:1 complex which is only 5% smaller than that in the 1:1 complex is well reproduced by the *ab initio* interaction energies, and that an even better agreement is found for the calculated complexation enthalpies in LKr.

## 3. Experimental

The samples of benzene (>99%), toluene (99.8%) and trifluoroiodomethane (CF_3_I, 99%) were purchased from Sigma Aldrich (St. Louis, MO, USA). The samples of chlorotrifluoromethane (CF_3_Cl, 99%) and bromotrifluoromethane (CF_3_Br, 99%) were obtained from Praxair (Danbury, CT, USA) and Pfaltz & Bauer (Waterbury, CT, USA), respectively. All samples were used without further purification. The solvent gas, krypton, had a stated purity of 99.9998% and was supplied by Air Liquide (Paris, France).

Infrared spectra were recorded using a Bruker IFS 66v Fourier transform spectrometer. A Globar source was used in combination with a Ge/KBr beam splitter and a LN_2_-cooled broad band MCT detector. The interferograms were averaged over 500 scans, Blackmann-Harris three-term apodized and Fourier transformed to yield spectra with a resolution of 0.5 cm^−1^. The experimental set-up to study the solutions in liquid noble gases has been described in detail before [31,32]. A liquid cell equipped with wedged Si windows and with a path length of 1 cm was used to record the spectra.

Raman spectra were recorded on a Trivista 557 spectrometer consisting of a double *f* = 50 cm monochromator equipped with a 2000 lines mm^−1^ grating and a *f* = 70 cm spectrograph using a 2400 lines mm^−1^ grating and a back-end illuminated LN_2_ cooled CCD detector. The spectra were recorded using the 514.5 nm line of a Spectra-Physics argon ion laser, 2017-Ar S/N 1665, with the power of the incident laser beam set to 0.8 W. Frequencies were calibrated using Ne emission lines and are expected to be accurate to 0.5 cm^−1^. The entrance slit to the spectrograph was selected to result in full widths at half-height of the Ne calibration lines between 0.4 and 0.5 cm^−1^. The experimental set-up, which includes a liquid cell equipped with four quartz windows at right angles, and the filling procedures have been described in detail before [33].

Equilibrium geometries and harmonic vibrational frequencies of monomers and complexes were obtained from *ab initio* calculations at the MP2/aug-cc-pVDZ(-PP) level using Gaussian09 [34]. A more reliable complexation energy was derived from a single point *ab initio* calculation at the MP2/aug-cc-pVTZ(-PP) level. During all calculations, corrections for BSSE [35] were accounted for using CP-corrected gradient techniques [36]. The standard aug-cc-pVD(T)Z basis sets were used for hydrogen, carbon, fluorine and chlorine. For bromine and iodine, the aug-cc-pVD(T)Z-PP basis sets with small-core energy-consistent relativistic pseudopotentials were used. The aug-cc-pVD(T)Z-PP basis sets used were downloaded from the EMSL basis set library [37].

To estimate the complexation enthalpies in the vapor phase and in the cryosolutions, corrections for zero-point vibrational and thermal influences were accounted for, using statistical thermodynamical calculations [38]. Corrections for solvent influences were estimated by using Monte Carlo Free Energy Perturbation (MC-FEP) simulations [39].

## 4. Conclusions

In this study, infrared and Raman spectroscopic data, illustrating the formation of C-X⋯π bonded complexes between the trifluorohalomethanes CF_3_X (with X = Br, I) and the aromatic model compounds benzene and toluene in liquid krypton, was presented. For all complexes observed, information on the relative stability in the cryosolutions was obtained by performing temperature studies. The resulting values for the complexation enthalpies are −6.5(3) kJ mol^−1^ for CF_3_Br·benzene, −7.6(2) kJ mol^−1^ for CF_3_I·benzene, −6.2(5) kJ mol^−1^ for CF_3_Br·toluene and −7.4(5) kJ mol^−1^ for CF_3_I·toluene. For the mixed solutions containing CF_3_I and benzene, a weak feature due to the induced ring breathing mode of benzene was observed in the infrared spectrum. Infrared and Raman experiments, supported by *ab initio* calculations, also revealed the formation of a 2:1 complex in which two CF_3_I molecules are bonded to a single molecule benzene. The experimental complexation enthalpy for this complex is −14.5(9) kJ mol^−1^. This value is only slightly smaller than twice the value obtained for the corresponding 1:1 complex and suggests that in the 2:1 complex only a weak anti-cooperative effect is present. Comparison of the experimental complexation enthalpies with the theoretical data derived from the *ab initio* calculations shows that for all complexes studied, the *ab initio* calculations lead to a significant overestimation of the complexation energies and enthalpies. The reason for this phenomenon is not yet understood.

## Supplementary Materials

Supplementary materials can be accessed at: http://www.mdpi.com/1420-3049/18/6/6829/s1.
