# Supplementary material for: Exploring the C-X…π Halogen Bonding Motif: An Infrared and Raman Study of the Complexes of CF3X (X = Cl, Br and I) with the Aromatic Model Compounds Benzene and Toluene
Source: Molecules. 2013 Jun 10;18(6):6829–51. doi: 10.3390/molecules18066829 (PMC6270472; doi:10.3390/molecules18066829)
Supplement: Supplementary file 1 [file molecules-18-06829-s001.pdf]

Supplementary Materials

Table S1. MP2/aug-cc-pVDZ vibrational frequencies, in cm^{-1}, and infrared intensities, in km mol^{-1}, for CF_3Cl•benzene.

Mode	$\nu_{monomer}$	IR Intensity	$\nu_{complex}$	IR Intensity	$\Delta\nu$
CF_3Cl					
ν_1 (A_1)	1097.0	469.7	1098.8	558.7	1.8
ν_2 (A_1)	765.0	24.5	763.2	21.0	−1.8
ν_3 (A_1)	480.3	0.1	481.5	1.1	1.2
ν_4 (E)	1192.2	282.1	1182.6	214.4	−9.6
ν_5 (E)	546.0	1.4	545.5	1.4	−0.5
ν_6 (E)	347.2	0.002	348.1	0.01	0.9
benzene					
ν_1 (A_{1g})	3238.7	0.0	3239.1	0.2	0.3
ν_2 (A_{1g})	1007.1	0.0	1006.3	1.0	−0.8
ν_3 (A_{2g})	1322.6	0.0	1321.9	0.0	−0.6
ν_4 (A_{2u})	678.4	115.9	680.6	128.7	2.2
ν_5 (B_{1u})	3202.1	0.0	3202.9	0.001	0.8
ν_6 (B_{1u})	969.6	0.0	969.1	0.0	−0.5
ν_7 (B_{2g})	904.4	0.0	904.5	0.0004	0.1
ν_8 (B_{2g})	577.4	0.0	573.4	0.001	−4.0
ν_9 (B_{2u})	1473.8	0.0	1476.6	0.003	2.8
ν_{10} (B_{2u})	1155.3	0.0	1155.4	0.0	0.1
ν_{11} (E_{1g})	845.5	0.0	847.3	0.2	1.8
ν_{12} (E_{1u})	3229.1	28.3	3229.6	22.5	0.5
ν_{13} (E_{1u})	1469.3	5.4	1468.0	6.6	−1.3
ν_{14} (E_{1u})	1052.0	5.2	1051.5	4.4	−0.5
ν_{15} (E_{2g})	3213.0	0.0	3213.7	0.003	0.8
ν_{16} (E_{2g})	1624.5	0.0	1622.5	0.004	−2.0
ν_{17} (E_{2g})	1183.1	0.0	1183.1	49.4	−0.1
ν_{18} (E_{2g})	594.9	0.0	594.2	0.001	−0.8
ν_{19} (E_{2u})	927.1	0.0	927.7	0.004	0.7
ν_{20} (E_{2u})	394.8	0.0	393.8	0.002	−1.1

Notes:Van der Waals vibrations: 53.7 cm^{-1}, 0.1 km mol^{-1}; 40.2 cm^{-1}, 0.001 km mol^{-1}; 38.3 cm^{-1}, 0.008 km mol^{-1}; 17.3 cm^{-1}, 0.02 km mol^{-1}; 9.8 cm^{-1}, 0.01 km mol^{-1}; 2.2 cm^{-1}, 0.0002 km mol^{-1}.

Table S2. MP2/aug-cc-pVDZ vibrational frequencies, in cm^{-1}, and infrared intensities, in km mol^{-1}, for CF_3Br·benzene.

Mode	$\nu_{monomer}$	IR Intensity	$\nu_{complex}$	IR Intensity	$\Delta\nu$
CF_3Br					
ν_1 (A_1)	1078.6	496.1	1080.0	581.9	1.4
ν_2 (A_1)	739.4	29.7	736.7	26.1	−2.7
ν_3 (A_1)	361.5	0.04	360.4	1.0	−1.1
ν_4 (E)	1178.8	254.7	1169.8	243.6	−9.0
ν_5 (E)	531.3	0.9	530.5	1.0	−0.8
ν_6 (E)	305.8	0.01	306.3	0.01	0.5
benzene					
ν_1 (A_{1g})	3238.7	0.0	3239.3	0.4	0.6
ν_2 (A_{1g})	1007.1	0.0	1005.9	1.6	−1.2
ν_3 (A_{2g})	1322.6	0.0	1321.4	0.0002	−1.2
ν_4 (A_{2u})	678.4	115.9	681.9	133.4	3.5
ν_5 (B_{1u})	3202.1	0.0	3203.4	0.004	1.3
ν_6 (B_{1u})	969.6	0.0	968.5	0.0002	−1.1
ν_7 (B_{2g})	904.4	0.0	904.6	0.004	0.2
ν_8 (B_{2g})	577.4	0.0	571.1	0.01	−6.4
ν_9 (B_{2u})	1473.8	0.0	1476.9	0.02	3.1
ν_{10} (B_{2u})	1155.3	0.0	1155.6	0.0004	0.3
ν_{11} (E_{1g})	845.5	0.0	848.1	0.5	2.6
ν_{12} (E_{1u})	3229.1	28.3	3229.9	20.3	0.8
ν_{13} (E_{1u})	1469.3	5.4	1467.3	7.0	−2.0
ν_{14} (E_{1u})	1052.0	5.2	1051.2	4.5	−0.8
ν_{15} (E_{2g})	3213.0	0.0	3214.2	0.008	1.2
ν_{16} (E_{2g})	1624.5	0.0	1621.7	0.02	−2.8
ν_{17} (E_{2g})	1183.1	0.0	1183.0	0.1	−0.1
ν_{18} (E_{2g})	594.9	0.0	593.9	0.003	−1.1
ν_{19} (E_{2u})	927.1	0.0	928.1	0.02	1.0
ν_{20} (E_{2u})	394.8	0.0	393.3	0.01	−1.5

Notes:Van der Waals vibrations: 57.7 cm^{-1}, 0.4 km mol^{-1}; 40.0 cm^{-1}, 0.01 km mol^{-1}; 38.4 cm^{-1}, 0.01 km mol^{-1}; 20.3 cm^{-1}, 0.02 km mol^{-1}; 8.7 cm^{-1}, 0.01 km mol^{-1}; 1.0 cm^{-1}, 0.0001 km mol^{-1}.

Table S3. MP2/aug-cc-pVDZ vibrational frequencies, in cm^{-1}, and infrared intensities, in km mol^{-1}, for CF_3I·benzene.

Mode	$\nu_{monomer}$	IR Intensity	$\nu_{complex}$	IR Intensity	$\Delta\nu$
CF_3I					
ν_1 (A_1)	1060.0	546.9	1063.4	625.2	3.5
ν_2 (A_1)	722.3	34.4	719.6	27.7	−2.8
ν_3 (A_1)	295.1	0.3	293.3	2.6	−1.8
ν_4 (E)	1162.0	227.6	1151.8	222.2	−10.2
ν_5 (E)	518.9	0.5	517.9	0.7	−1.0
ν_6 (E)	269.1	0.03	269.5	0.003	0.3
benzene					
ν_1 (A_{1g})	3238.7	0.0	3239.6	0.9	0.9
ν_2 (A_{1g})	1007.1	0.0	1005.3	3.1	−1.8
ν_3 (A_{2g})	1322.6	0.0	1320.6	0.001	−2.0
ν_4 (A_{2u})	678.4	115.9	683.5	141.6	5.1
ν_5 (B_{1u})	3202.1	0.0	3203.7	0.01	1.6
ν_6 (B_{1u})	969.6	0.0	967.6	0.001	−2.0
ν_7 (B_{2g})	904.4	0.0	904.4	0.03	−0.1
ν_8 (B_{2g})	577.4	0.0	566.7	0.03	−10.7
ν_9 (B_{2u})	1473.8	0.0	1477.3	0.05	3.5
ν_{10} (B_{2u})	1155.3	0.0	1155.8	0.01	0.5
ν_{11} (E_{1g})	845.5	0.0	849.0	1.1	3.5
ν_{12} (E_{1u})	3229.1	28.3	3230.2	17.4	1.1
ν_{13} (E_{1u})	1469.3	5.4	1466.4	7.4	−2.9
ν_{14} (E_{1u})	1052.0	5.2	1050.8	6.5	−1.2
ν_{15} (E_{2g})	3213.0	0.0	3214.6	0.02	1.7
ν_{16} (E_{2g})	1624.5	0.0	1620.6	0.1	−3.8
ν_{17} (E_{2g})	1183.1	0.0	1182.9	0.05	−0.2
ν_{18} (E_{2g})	594.9	0.0	593.5	0.01	−1.5
ν_{19} (E_{2u})	927.1	0.0	928.3	0.1	1.2
ν_{20} (E_{2u})	394.8	0.0	392.5	0.03	−2.3

Notes:Van der Waals vibrations: 62.3 cm^{-1}, 1.1 km mol^{-1}; 41.8 cm^{-1}, 0.01 km mol^{-1}; 39.3 cm^{-1}, 0.03 km mol^{-1}; 22.2 cm^{-1}, 0.03 km mol^{-1}; 8.9 cm^{-1}, 0.004 km mol^{-1}; 0.1 cm^{-1}, 0.0 km mol^{-1}.

Table S4. MP2/aug-cc-pVDZ vibrational frequencies, in cm^{-1}, and infrared intensities, in km mol^{-1}, for CF_3Cl·toluene.

Mode	$\nu_{monomer}$	IR Intensity	$\nu_{complex}$	IR Intensity	$\Delta\nu$
CF_3Cl					
ν_1 (A_1)	1097.0	469.7	1097.9	566.5	1.0
ν_2 (A_1)	765.0	24.5	762.7	21.3	−2.2
ν_3 (A_1)	480.3	0.1	481.2	1.6	0.9
ν_4 (E)	1192.2	282.1	1183.0	260.4	−9.2
ν_5 (E)	546.0	1.4	545.4	1.5	−0.5
ν_6 (E)	347.2	0.002	348.1	0.01	0.9
toluene					
ν_1 (A_1)	3234.3	12.0	3235.0	10.4	0.8
ν_2 (A_1)	3212.1	3.8	3213.1	2.9	0.9
ν_3 (A_1)	3194.5	7.2	3196.6	6.1	2.1
ν_4 (A_1)	1641.4	5.9	1639.0	5.3	−2.4
ν_5 (A_1)	1497.5	10.5	1495.8	11.1	−1.7
ν_6 (A_1)	1238.1	0.3	1237.8	0.1	−0.3
ν_7 (A_1)	1187.3	0.1	1187.2	0.2	−0.1
ν_8 (A_1)	1041.5	3.2	1041.2	2.1	−0.3
ν_9 (A_1)	992.0	0.3	992.4	0.4	0.5
ν_{10} (A_1)	790.6	0.2	790.3	0.8	−0.2
ν_{11} (A_1)	512.8	0.4	513.1	0.5	0.2
ν_{12} (A_2)	931.8	0.01	933.2	0.01	1.4
ν_{13} (A_2)	837.2	0.001	839.1	0.1	1.9
ν_{14} (A_2)	400.4	0.01	397.9	0.01	−2.5
ν_{15} (B_1)	914.8	0.004	916.6	0.03	1.9
ν_{16} (B_1)	878.9	0.3	881.4	0.8	2.5
ν_{17} (B_1)	721.2	77.8	722.5	82.0	1.3
ν_{18} (B_1)	609.8	1.7	610.0	2.5	0.2
ν_{19} (B_1)	458.5	8.3	457.7	10.0	−0.9
ν_{20} (B_1)	210.1	2.0	211.5	3.2	1.3
ν_{21} (B_2)	3221.2	26.6	3222.0	21.3	0.9
ν_{22} (B_2)	3198.6	6.1	3198.1	4.7	−0.5
ν_{23} (B_2)	1619.2	0.03	1616.6	0.01	−2.6
ν_{24} (B_2)	1477.0	1.5	1478.3	3.3	1.3
ν_{25} (B_2)	1436.4	0.8	1434.0	1.3	−2.3
ν_{26} (B_2)	1314.5	0.2	1311.9	0.1	−2.6
ν_{27} (B_2)	1162.8	0.003	1163.0	0.01	0.2
ν_{28} (B_2)	1100.1	5.7	1100.0	13.9	−0.1
ν_{29} (B_2)	614.0	0.1	612.8	0.1	−1.2
ν_{30} (B_2)	337.3	0.3	337.9	0.2	0.7
ν_{31} (A_1)	3061.1	27.5	3057.5	24.1	−3.7
ν_{32} (A_1)	1397.0	0.4	1397.3	0.9	0.2
ν_{33} (A_2)	43.2	0.1	40.2	0.01	−3.0
ν_{34} (E)	3154.4	12.5	3153.1	12.6	−1.3
ν_{35} (E)	1485.6	7.8	1484.0	6.5	−1.6
ν_{36} (E)	1021.2	1.9	1018.2	4.4	−3.0

Notes:Van der Waals vibrations: 54.9 cm^{-1}, 0.1 km mol^{-1}; 32.7 cm^{-1}, 0.04 km mol^{-1}; 19.8 cm^{-1}, 0.05 km mol^{-1}; 12.1 cm^{-1}, 0.02 km mol^{-1}; 3.6 cm^{-1}, 0.1 km mol^{-1}; 0.7 cm^{-1}, 0.03 km mol^{-1}.

Table S5. MP2/aug-cc-pVDZ vibrational frequencies, in cm^{-1}, and infrared intensities, in km mol^{-1}, for CF_3Br·toluene.

Mode	$\nu_{monomer}$	IR Intensity	$\nu_{complex}$	IR Intensity	$\Delta\nu$
CF_3Br					
ν_1 (A_1)	1078.6	496.1	1079.6	596.5	1.0
ν_2 (A_1)	739.4	29.7	736.3	23.4	−3.1
ν_3 (A_1)	361.5	0.04	359.5	1.3	−2.0
ν_4 (E)	1178.8	254.7	1169.7	240.9	−9.1
ν_5 (E)	531.3	0.9	530.4	1.0	−0.9
ν_6 (E)	305.8	0.01	306.2	0.01	0.5
toluene					
ν_1 (A_1)	3234.3	12.0	3235.6	9.9	1.3
ν_2 (A_1)	3212.1	3.8	3213.6	2.4	1.5
ν_3 (A_1)	3194.5	7.2	3196.6	5.6	2.2
ν_4 (A_1)	1641.4	5.9	1638.3	4.9	−3.1
ν_5 (A_1)	1497.5	10.5	1495.7	10.0	−1.8
ν_6 (A_1)	1238.1	0.3	1237.8	0.1	−0.3
ν_7 (A_1)	1187.3	0.1	1187.2	0.2	−0.1
ν_8 (A_1)	1041.5	3.2	1040.9	2.6	−0.6
ν_9 (A_1)	992.0	0.3	991.5	0.4	−0.4
ν_{10} (A_1)	790.6	0.2	789.7	0.6	−0.9
ν_{11} (A_1)	512.8	0.4	512.4	0.4	−0.5
ν_{12} (A_2)	931.8	0.01	933.1	0.1	1.3
ν_{13} (A_2)	837.2	0.001	840.0	0.4	2.8
ν_{14} (A_2)	400.4	0.01	398.4	0.1	−1.9
ν_{15} (B_1)	914.8	0.004	916.2	0.03	1.4
ν_{16} (B_1)	878.9	0.3	881.7	1.4	2.8
ν_{17} (B_1)	721.2	77.8	723.4	87.8	2.2
ν_{18} (B_1)	609.8	1.7	605.7	2.0	−4.1
ν_{19} (B_1)	458.5	8.3	458.2	11.0	−0.3
ν_{20} (B_1)	210.1	2.0	214.4	3.9	4.3
ν_{21} (B_2)	3221.2	26.6	3222.5	19.4	1.4
ν_{22} (B_2)	3198.6	6.1	3198.7	4.4	0.1
ν_{23} (B_2)	1619.2	0.03	1616.2	0.03	−3.0
ν_{24} (B_2)	1477.0	1.5	1478.9	3.2	1.9
ν_{25} (B_2)	1436.4	0.8	1434.6	1.6	−1.7
ν_{26} (B_2)	1314.5	0.2	1313.4	0.2	−1.2
ν_{27} (B_2)	1162.8	0.003	1163.4	0.1	0.6
ν_{28} (B_2)	1100.1	5.7	1099.9	5.1	−0.3
ν_{29} (B_2)	614.0	0.1	612.8	0.04	−1.2
ν_{30} (B_2)	337.3	0.3	337.2	0.3	−0.1
ν_{31} (A_1)	3061.1	27.5	3059.2	22.6	−1.9
ν_{32} (A_1)	1397.0	0.4	1397.3	1.2	0.3
ν_{33} (A_2)	43.2	0.1	47.2	0.1	4.0
ν_{34} (E)	3154.4	12.5	3153.9	11.9	−0.5
ν_{35} (E)	1485.6	7.8	1484.4	8.2	−1.1
ν_{36} (E)	1021.2	1.9	1020.1	4.4	−1.0

Notes:Van der Waals vibrations: 57.8 cm^{-1}, 0.3 km mol^{-1}; 39.6 cm^{-1}, 0.1 km mol^{-1}; 31.8 cm^{-1}, 0.01 km mol^{-1}; 21.1 cm^{-1}, 0.05 km mol^{-1}; 11.6 cm^{-1}, 0.02 km mol^{-1}; 1.1 cm^{-1}, 0.003 km mol^{-1}.

Table S6. MP2/aug-cc-pVDZ vibrational frequencies, in cm^{-1}, and infrared intensities, in km mol^{-1}, for CF_3I·toluene.

Mode	$\nu_{monomer}$	IR Intensity	$\nu_{complex}$	IR Intensity	$\Delta\nu$
CF_3I					
ν_1 (A_1)	1060.0	546.9	1063.4	640.9	3.5
ν_2 (A_1)	722.3	34.4	719.0	43.1	−3.4
ν_3 (A_1)	295.1	0.3	292.3	3.2	−2.8
ν_4 (E)	1162.0	227.6	1151.1	220.2	−10.9
ν_5 (E)	518.9	0.5	517.7	0.7	−1.2
ν_6 (E)	269.1	0.03	269.3	0.004	0.2
toluene					
ν_1 (A_1)	3234.3	12.0	3236.2	9.2	1.9
ν_2 (A_1)	3212.1	3.8	3214.2	1.9	2.1
ν_3 (A_1)	3194.5	7.2	3197.4	5.0	2.9
ν_4 (A_1)	1641.4	5.9	1637.1	4.6	−4.3
ν_5 (A_1)	1497.5	10.5	1495.1	9.1	−2.3
ν_6 (A_1)	1238.1	0.3	1237.6	0.2	−0.5
ν_7 (A_1)	1187.3	0.1	1187.1	0.3	−0.2
ν_8 (A_1)	1041.5	3.2	1040.7	2.2	−0.8
ν_9 (A_1)	992.0	0.3	991.2	0.7	−0.8
ν_{10} (A_1)	790.6	0.2	789.1	0.6	−1.5
ν_{11} (A_1)	512.8	0.4	511.9	0.4	−0.9
ν_{12} (A_2)	931.8	0.01	933.5	0.3	1.6
ν_{13} (A_2)	837.2	0.001	841.4	1.1	4.2
ν_{14} (A_2)	400.4	0.01	398.2	0.2	−2.2
ν_{15} (B_1)	914.8	0.004	916.6	0.04	1.9
ν_{16} (B_1)	878.9	0.3	882.9	2.5	3.9
ν_{17} (B_1)	721.2	77.8	724.7	74.1	3.5
ν_{18} (B_1)	609.8	1.7	602.3	1.7	−7.5
ν_{19} (B_1)	458.5	8.3	457.5	11.7	−1.0
ν_{20} (B_1)	210.1	2.0	215.6	5.5	5.4
ν_{21} (B_2)	3221.2	26.6	3223.0	16.8	1.8
ν_{22} (B_2)	3198.6	6.1	3198.5	3.7	−0.1
ν_{23} (B_2)	1619.2	0.03	1615.3	0.1	−3.9
ν_{24} (B_2)	1477.0	1.5	1478.9	3.4	1.9
ν_{25} (B_2)	1436.4	0.8	1434.1	1.8	−2.3
ν_{26} (B_2)	1314.5	0.2	1313.3	0.3	−1.2
ν_{27} (B_2)	1162.8	0.003	1163.8	0.0	1.0
ν_{28} (B_2)	1100.1	5.7	1099.7	5.0	−0.5
ν_{29} (B_2)	614.0	0.1	612.4	0.02	−1.5
ν_{30} (B_2)	337.3	0.3	336.9	0.2	−0.4
ν_{31} (A_1)	3061.1	27.5	3060.0	20.5	−1.1
ν_{32} (A_1)	1397.0	0.4	1397.4	1.6	0.4
ν_{33} (A_2)	43.2	0.1	56.1	0.2	12.9
ν_{34} (E)	3154.4	12.5	3154.5	11.1	0.1
ν_{35} (E)	1485.6	7.8	1484.3	10.0	−1.3
ν_{36} (E)	1021.2	1.9	1020.3	6.1	−0.9

Notes:Van der Waals vibrations: 61.3 cm^{-1}, 1.0 km mol^{-1}; 42.3 cm^{-1}, 0.05 km mol^{-1}; 35.2 cm^{-1}, 0.03 km mol^{-1}; 23.6 cm^{-1}, 0.04 km mol^{-1}; 13.6 cm^{-1}, 0.02 km mol^{-1}; 0.9 cm^{-1}, 0.002 km mol^{-1}.

Table S7. Comparison of the experimentally observed frequencies of toluene in LKr at 120 K with literature data [a] for the vapor and liquid phases and with the harmonic vibrational frequencies derived at the MP2/aug-cc-PVDZ and MP2/aug-cc-pVTZ levels.

Assign	Sym	Gas	Liquid		LKr		Calc	
		IR	Raman	IR	Raman	IR	MP2/DZ	MP2/TZ
ν_{21}	B_2	3096		3086.4		3092.9	3221.2	3212.9
ν_{1}	A_1	3073	3065	3062.1	3072.5	3072.2	3234.3	3230.7
ν_{2}	A_1		3055	3055	3060.7	3061.1	3212.1	3202.8
ν_{3}	A_1		3038		3045.5	3046.2	3194.5	3180.5
ν_{22}	B_2	3035		3027.0		3032.4	3198.6	3185.0
ν_{34}	E	2960–2920		2950	2954.0	2954.7	3154.4	3153.5
ν_{31}	A_1	2960–2920	2920	2919.9	2923.5	2923.1	3061.1	3074.7
ν_{4}	A_1	1609	1604	1604.6	1608.0	1607.8	1641.4	1657.8
ν_{23}	B_2		1585	1586.7	1567.5	1586.9	1619.2	1635.8
ν_{5}	A_1	1500		1495.7	1498.2	1497.4	1497.5	1531.0
ν_{35}	E	1463		1460.3		1466.9	1485.6	1519.1
ν_{24}	B_2		1442		1440.9	1441.4	1477.0	1482.9
ν_{32}	A_1	1384	1378	1378.9	1380.5	1380.1	1397.0	1423.4
ν_{25}	B_2		1332	1332.0	1332.4	1331.8	1436.4	1462.8
ν_{26}	B_2	1310		1312.7	1310.9	1310.8	1314.5	1345.0
ν_{6}	A_1	1213	1210	1210.2	1212.1	1211.7	1238.1	1255.0
ν_{7}	A_1	1179	1179	1178.6	1179.4	1179.0	1187.3	1198.6
ν_{27}	B_2		1156	1155.9	1155.9	1155.6	1162.8	1171.4
ν_{28}	B_2	1082		1081.4	1082.3	1081.6	1100.1	1114.2
ν_{36}	E			1041.4		1040.7	1021.2	1045.0
ν_{8}	A_1	1032	1031	1030.1	1032.0	1031.6	1041.5	1058.2
ν_{9}	A_1		1003	1002.3	1002.9	1002.4	992.0	1029.1
ν_{15}	B_1			980.7			914.8	1013.0
ν_{12}	A_2			966.4			931.8	999.0
ν_{16}	B_1	894		895.4	894.3	893.9	878.9	933.4
ν_{13}	A_2		842	842.7	841.2		837.2	875.3
ν_{10}	A_1	786	786	785.6	786.8	786.4	790.6	807.4
ν_{17}	B_1	730		729.9	729.2	728.6	721.2	760.0
ν_{18}	B_1	694		694.8		694.5	609.8	713.4
ν_{29}	B_2		622	622.0	624.5		614.0	624.1
ν_{11}	A_1	520	521	521.0	521.7		512.8	522.4
ν_{19}	B_1	462		464.4	464.0	463.8	458.5	478.4
ν_{14}	A_2						400.4	412.6
ν_{30}	B_2		346		345.8		337.3	338.0
ν_{20}	B_1		217		217.5		210.1	216.7
ν_{33}	A_2						43.2	73.9

[a] J. E. Bertie, Y. Apelblat and C. D. Keefe. Infrared intensities of liquids XXV: Dielectric constants, molar polarizabilities and integrated intensities of liquid toluene at 25 °C between 4800 and 400 cm^{-1}. *Journal of Molecular Structure* **2005**, *750*, 78–93.
